# Implementing a trauma-informed approach in a tiered model of pediatric population mental health care: a pilot study in primary and secondary care

**DOI:** 10.1186/s12913-025-13356-7

**Published:** 2025-12-18

**Authors:** Caley Mikesell, Emily J. Blevins, Miranda Eschtruth, Katelyn Malvese, Mary Wallace, Yu Zhou, Younga H. Lee, Dana Allswede, Makiko Watanabe, Nancy Rotter, Archana Basu

**Affiliations:** 1 Department of Psychiatry, Massachusetts General Hospital, Boston, MA USA; 2 The Broad Trauma Initiative, Broad Institute of MIT and Harvard, Cambridge, MA USA; 3 Psychiatric & Neurodevelopmental Genetics Unit, Center for Genomic Medicine, Massachusetts General Hospital, Boston, MA USA

**Keywords:** Trauma-informed care, Pediatric mental health care, Population mental health, Integrated primary care, Trauma, Adversity, Emotion dysregulation, Prevalence

## Abstract

**Background:**

Childhood adversity and trauma are prevalent risk factors for the development of mental health conditions. This two-part paper describes the conceptual basis and pilot implementation of a tiered model of pediatric population mental health, highlighting the local socioecological context in which it was developed and the trauma-informed approach used.

**Methods:**

Using retrospective record review of three datasets from the primary and secondary care pediatric clinics of a large academic medical center, which were harmonized to cover the study period from July 1, 2023 to June 30, 2024, we conducted descriptive analyses of patients across three levels: pediatric primary care (*n* = 9535), an integrated primary care program, which embeds mental health clinicians in primary care (*n* = 267), and family-centered trauma-informed psychotherapies in secondary care (*n* = 63), designed to address emotion dysregulation in pre-adolescent children. Demographics and lifetime history of trauma and adversity (assessed with a comprehensive 19-item list coded based on standardized screeners) were assessed through electronic medical records.

**Results:**

Relative to the pediatric primary care population, more patients in the integrated primary care program and trauma-informed psychotherapies identified as White. Using our 19-item assessment, the lifetime prevalence of adversity or trauma was nearly universal among patients in the integrated primary care (94.4%) and trauma-informed psychotherapy (98.4%) programs. However, the lifetime prevalence of childhood adversities differed significantly across the two programs (integrated primary care: 76.8%; trauma-informed psychotherapy: 98.4%) when we assessed prevalence based only on the 10-item Adverse Childhood Experiences Questionnaire (Felitti VJ, Anda RF, Nordenberg D, Williamson DF, Spitz AM, Edwards V, Koss MP, Marks JS, Am J Prev Med 14:245–58, 1998). There was a higher prevalence of family and parent-related adversities in the trauma-informed psychotherapy program.

**Conclusions:**

Findings support the need for trauma-informed, population mental health approaches in pediatric care. Developmentally tailored, family-centered, transdiagnostic screening and interventions are essential. Study findings, including gaps in programmatic fiscal sustainability, suggest avenues for policy reform to support and scale trauma-informed programs like ours. Programs seeking to implement trauma-informed approaches should leverage implementation and participatory research to ensure effectiveness and equitable accessibility for patients of diverse identities.

**Supplementary Information:**

The online version contains supplementary material available at 10.1186/s12913-025-13356-7.

## Background

Mounting evidence demonstrates that childhood adversity and trauma are prevalent risk factors for the development of a range of mental [1–3] and physical [4–6] health problems. In the United States (US), about two-thirds of adults report a history of Adverse Childhood Experiences (ACEs) [4, 7], with epidemiologic studies suggesting that 20% of children experience at least one ACE by age three [8] and 60–70% of youth are exposed to a range of potentially traumatic events by age 17 [9–11]. Youth with marginalized identities, including those who are Black or Hispanic, of low socioeconomic status, or living in states with high child poverty rates, face even higher exposure rates [9, 12–15]. Originally defined as experiences of child maltreatment (e.g., neglect, abuse) and household dysfunction (parental mental illness, divorce) [4], childhood adversity can also encompass community-level hardships (e.g., witnessing violence, experiencing discrimination; see [16]). Some forms of adversity align with the current Diagnostic and Statistical Manual of Mental Disorders (DSM-5-TR) definition of “Criterion A trauma” for the diagnosis of posttraumatic stress disorder—“actual or threatened death, serious injury, or sexual violence” [17]—whereas others, though deleterious, do not. In other words, while all traumatic experiences may be considered adversity, not all adverse experiences constitute trauma as defined by the DSM-5-TR. That said, both childhood adversity and trauma may increase susceptibility to the development of mental health conditions [18, 19] through diverse mechanisms (e.g., emotion dysregulation [20]). Notably, the onset of approximately one-third of all diagnosed mental health conditions can be attributed to childhood adversity [1–3]. Exposure to some types of early life adversity is also associated with more severe, persistent, and treatment-resistant symptoms, and may moderate treatment trajectories [21, 22]. Given their prevalence in the general population and broad sequelae across the lifespan, preventing and mitigating the effects of childhood adversity and trauma are foundational to promoting population health.

**Fig. 1:**
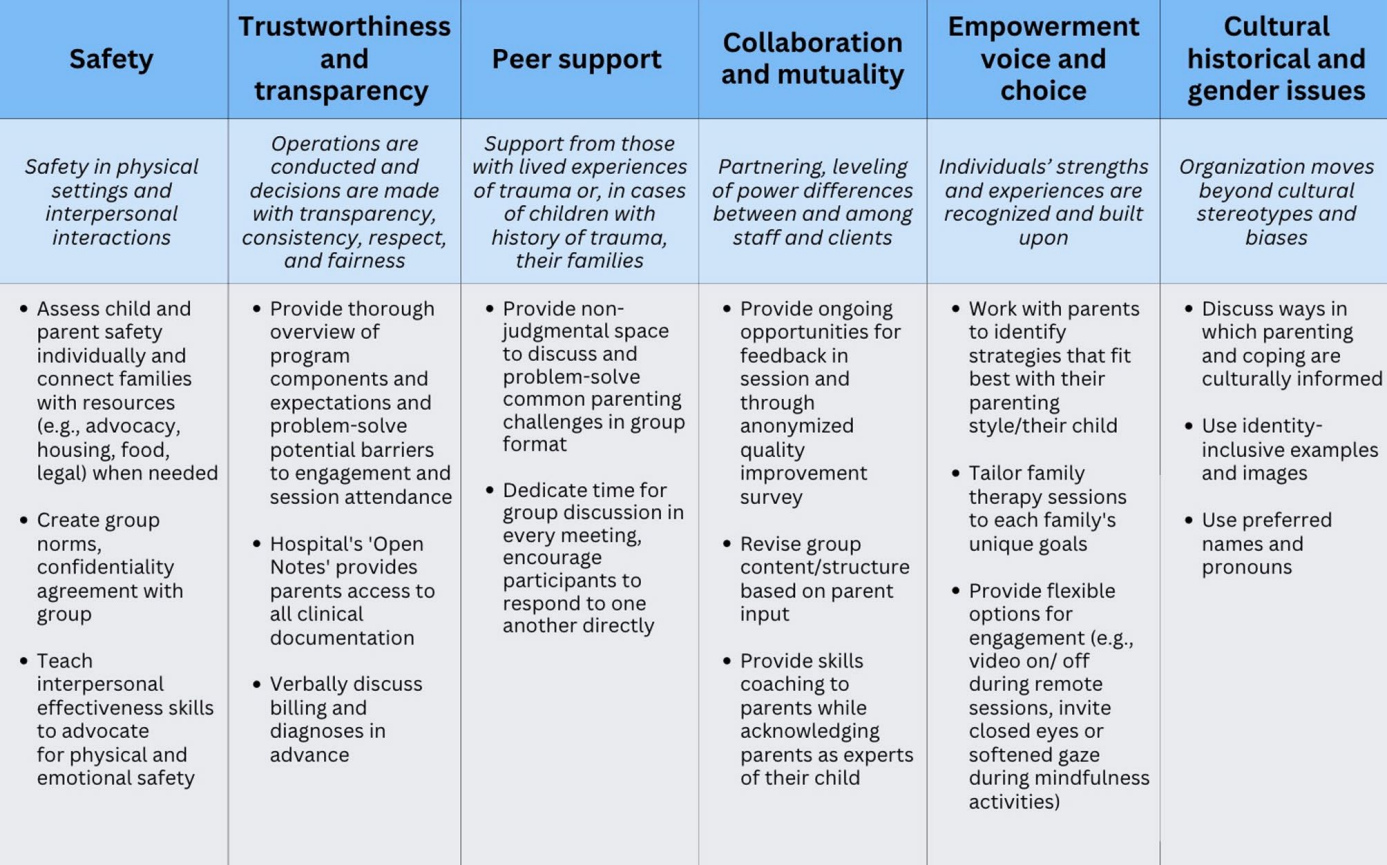
Integrating SAMHSA trauma-informed principles in psychotherapy interventions. Note: Based on trauma-informed care principles developed by SAMHSA [25]

Trauma-focused interventions for individuals are a critical component of the mental health care infrastructure; however, these are insufficient to optimize all survivors’ outcomes due to limited accessibility and the widespread impact of trauma and adversity [23–26]. Trauma-informed care shifts the focus from treatment protocols targeting specific diagnoses and Criterion A trauma sequelae (e.g., trauma-focused Cognitive Behavioral Therapy) to building responsive systems that emphasize the impact of service delivery on survivor outcomes, provider well-being, and effectiveness [25]. As defined by the Substance Abuse and Mental Health Services Administration [25], trauma-informed care is a framework through which individuals, programs, organizations, or systems conceptualize trauma and respond to survivors. Rather than a prescriptive model, this framework emphasizes adherence to trauma-informed care principles (see Fig. 1 [25]) and four assumptions: **Realize** the prevalence and outcomes associated with trauma; **Recognize** symptoms and other signs of trauma; **Respond** proactively by incorporating knowledge about trauma into procedures, policy, and structures; and **Resist** engaging in practices that could re-traumatize survivors [25]. Widely adopted in healthcare and educational settings, the framework emphasizes a socioecological perspective, acknowledging the impact of parenting, family, school, community, and broader systems on child development [26, 27]. Although research on trauma-informed approaches is mixed due in part to the heterogeneous operationalizations of the term [23, 28–31], SAMHSA’s framework is still considered evidence-informed because it is grounded in robust literature on the effects of trauma and adversity.

Efforts to expand trauma-informed care must address the inequities that underpin the broader pediatric mental health gap [32–34]. Approximately one in five US youth meet criteria for a mental health condition in a given year [35], yet only about half of those affected receive treatment [36, 37]. From a socioecological perspective, several multi-level factors impede equitable access [33, 38], including limited service availability, cost, insurance barriers, and prior negative experiences with providers [38–40]. Additional challenges, such as stigma [41, 42], discrimination [43], and cultural or linguistic incongruence of care and delivery models [44], further exacerbate disparities. State-level policy differences also affect cost, mental health parity law implementation, and, ultimately, mental health service use [32, 45, 46]. Failure to address these implementation and capacity challenges can result in the replication of existing inequities in new delivery models.

Population approaches are crucial to equitably meeting mental health needs and expanding trauma-informed care [26]. In particular, tiered models of care that incorporate integrated mental and physical health service delivery, evidence-based screening, and tailored referral pathways for specialized care may be particularly responsive to the diverse needs of pediatric populations, which vary based on developmental stage, individual risk and resilience factors, and trauma or adversity histories [26, 47–50]. Integrated primary care, wherein mental health clinicians (e.g., psychologists, social workers) form part of the primary care team, offers a well-established, evidence-based approach [49, 51–54] that could serve as a foundational component of a tiered model of pediatric mental health care. Research demonstrates benefits for patients across the lifespan, communities, and healthcare teams, suggesting such models improve mental health care capacity, equitable access, health behaviors, and patient outcomes, while also reducing stigma, cost, and clinical burden [49, 50, 52, 55–58]. In settings where most children access primary care (e.g., Massachusetts [59]), such models can facilitate population-level access to trauma-informed care, including whole-person assessments, mental health screening, and referral to appropriate secondary care interventions. That said, universal adversity and trauma screening (e.g., ACEs questionnaire) was developed as a tool for population-level surveillance rather than clinical decision-making for individual patients [60]. Indeed, universal screening has limited predictive value for identifying individual risk for future health problems [61–64], poses unique implementation challenges [65], and does not elucidate potential differences between trauma and adversity-related sequelae which may reflect differential mechanistic pathways, and correspondingly, require different treatment approaches [66, 67]. For this reason, universal screening for transdiagnostic processes, such as emotion dysregulation, that are associated with trauma and adversity [68–73] and which are risk factors for maladaptive behaviors and future psychopathology [71, 73–77], can be used as part of a trauma-informed approach in integrated primary care to facilitate early identification and targeted intervention. Taken together, evidence suggests tiered models of mental health care incorporating trauma-informed practices may be particularly well-suited to meeting the needs of pediatric populations. Additionally, further research is needed to understand how to best develop and implement such models to ensure effectiveness, equity, and scalability.

**Fig. 2:**
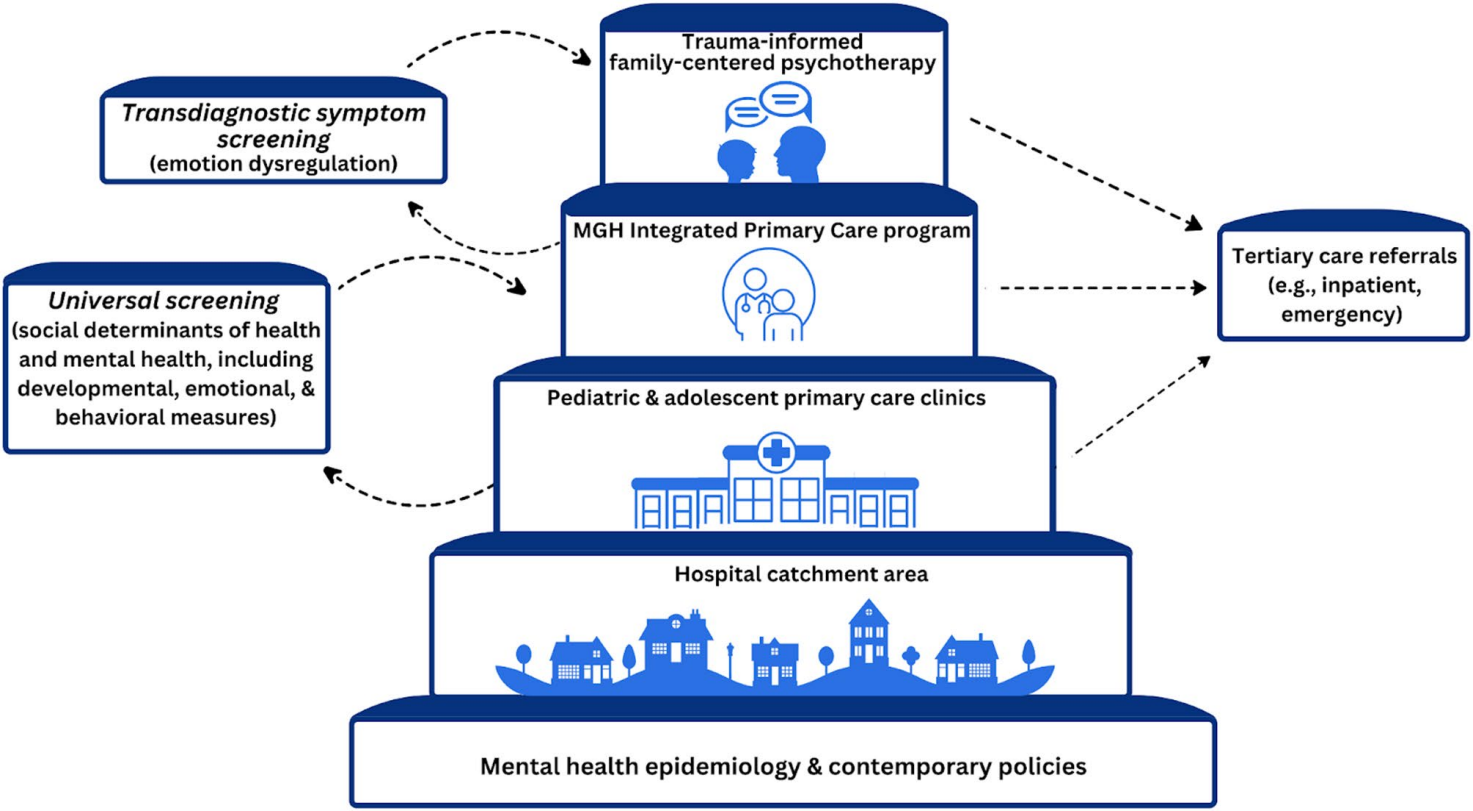
Socioecological contextualization of a trauma-informed approach to a tiered model of pediatric mental health care. Note: Adapted from Office of Health Equity, California Department of Public Health [78]

To address these gaps in the literature, this two-part paper aims to describe the conceptual basis and pilot implementation of a tiered model of pediatric mental health care in a large academic medical center. Our model consists of three increasingly specialized levels of care: (i) general pediatric and adolescent primary care clinics (infants–21 years), (ii) an integrated primary care program (infants–21 years), and (iii) trauma-informed outpatient psychotherapy interventions for preadolescent children (2–12 years) in a secondary care clinic. In Part I, we first provide a detailed description of the socioecological context in which our tiered model was implemented, and then describe the development of our trauma-informed, family-based screening strategies and interventions (Fig. 2 [78]). We provide a comprehensive description of our setting because socioecological context influences implementation effectiveness, ecological validity, and outcomes of interventions [33]. We hypothesized that a trauma-informed approach would be indicated in our model due to the probable prevalence of trauma and adversity. To test this hypothesis, in Part II, we first compare the demographic characteristics of patients across the three levels of care. Then, we compare the lifetime prevalence of adversity and trauma in patients in the integrated primary care program and trauma-informed psychotherapy interventions using retrospective record review. Finally, based on our descriptive findings, we outline future directions for trauma-informed clinical frameworks, considerations for policy makers to improve pediatric population mental health, and future directions for implementation research to evaluate our model.

## Methods

### Part I: Socioecological context and programmatic descriptions of a tiered model of pediatric mental health care

In this section, we contextualize and describe our tiered model of pediatric mental health care, developed and piloted within the Massachusetts General Hospital (MGH) hospital system in Boston, Massachusetts (see Fig. 2 [78]). Serving young people from birth through age 21, we refer to these programs as “pediatric” programs for concision hereafter. This tiered model was established to meet three primary aims: (i) reduce the clinical burden of addressing mental health needs in general pediatric and adolescent primary care (hereafter “pediatric primary care”), (ii) facilitate timely access to mental health services, and (iii) pilot targeted trauma-informed interventions to increase our clinical capacity in secondary care.

**Fig. 3:**
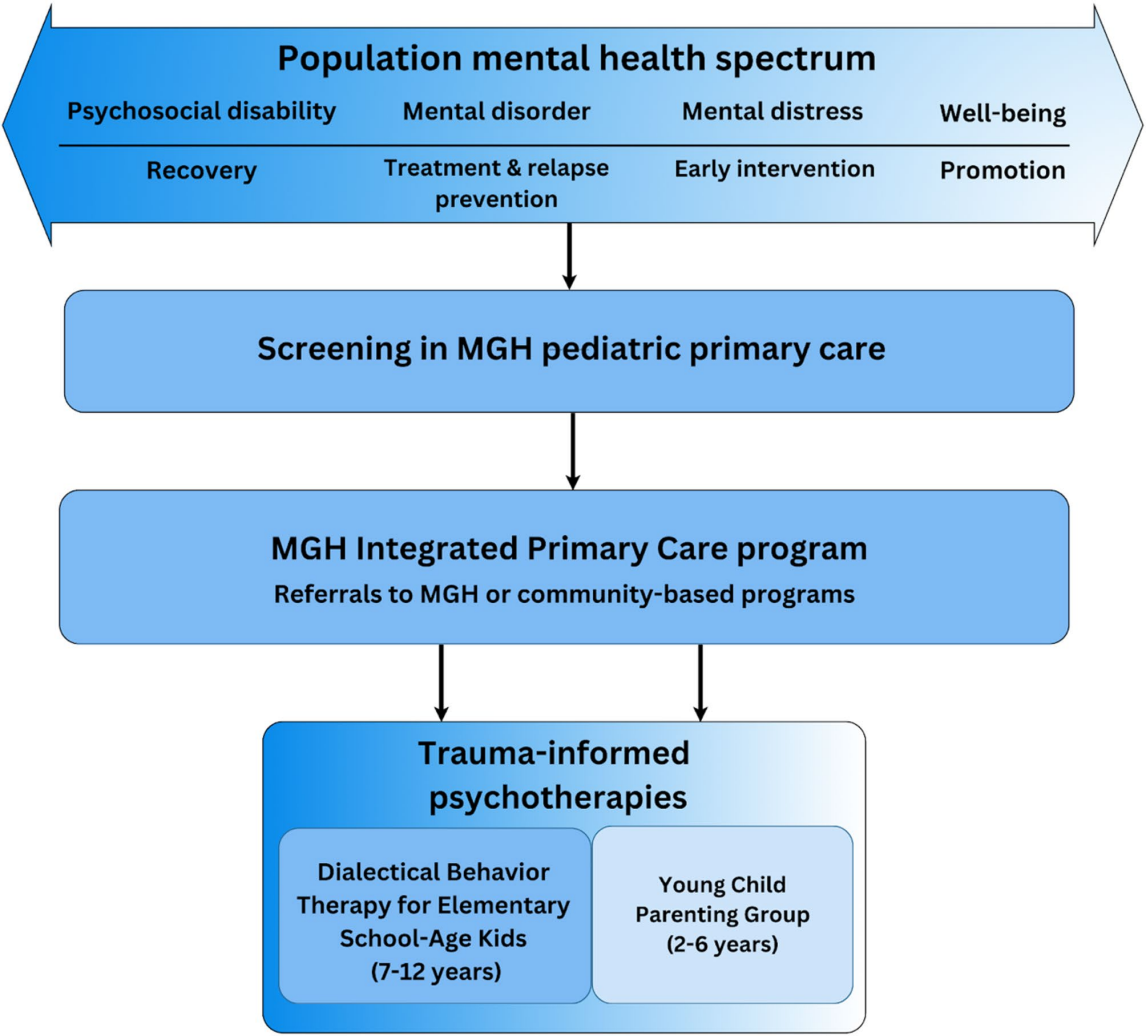
Pilot implementation of a trauma-informed, population mental health approach to a tiered model of pediatric mental health care

Beginning with the broadest level, we describe our pediatric primary care clinics as well as the socioecological context of the population they serve. Using a trauma-informed approach, we describe how this data informed our program development, including the establishment of new procedures and the successive tiers of care. Next, we provide the programmatic description of our second tier of care, the Massachusetts General Hospital Integrated Primary Care program (hereafter “MGH IPC”), including the family-centered, trauma-informed screening strategies used to identify a subset of patients within this program who may benefit from follow up in secondary care. Finally, we describe the conceptual basis and operationalization for two targeted trauma-informed psychotherapy interventions in our third tier, delivered within a child psychiatry outpatient clinic (see Fig. 3).

#### I. Socioecological context of pediatric primary care clinics

**Fig. 4:**
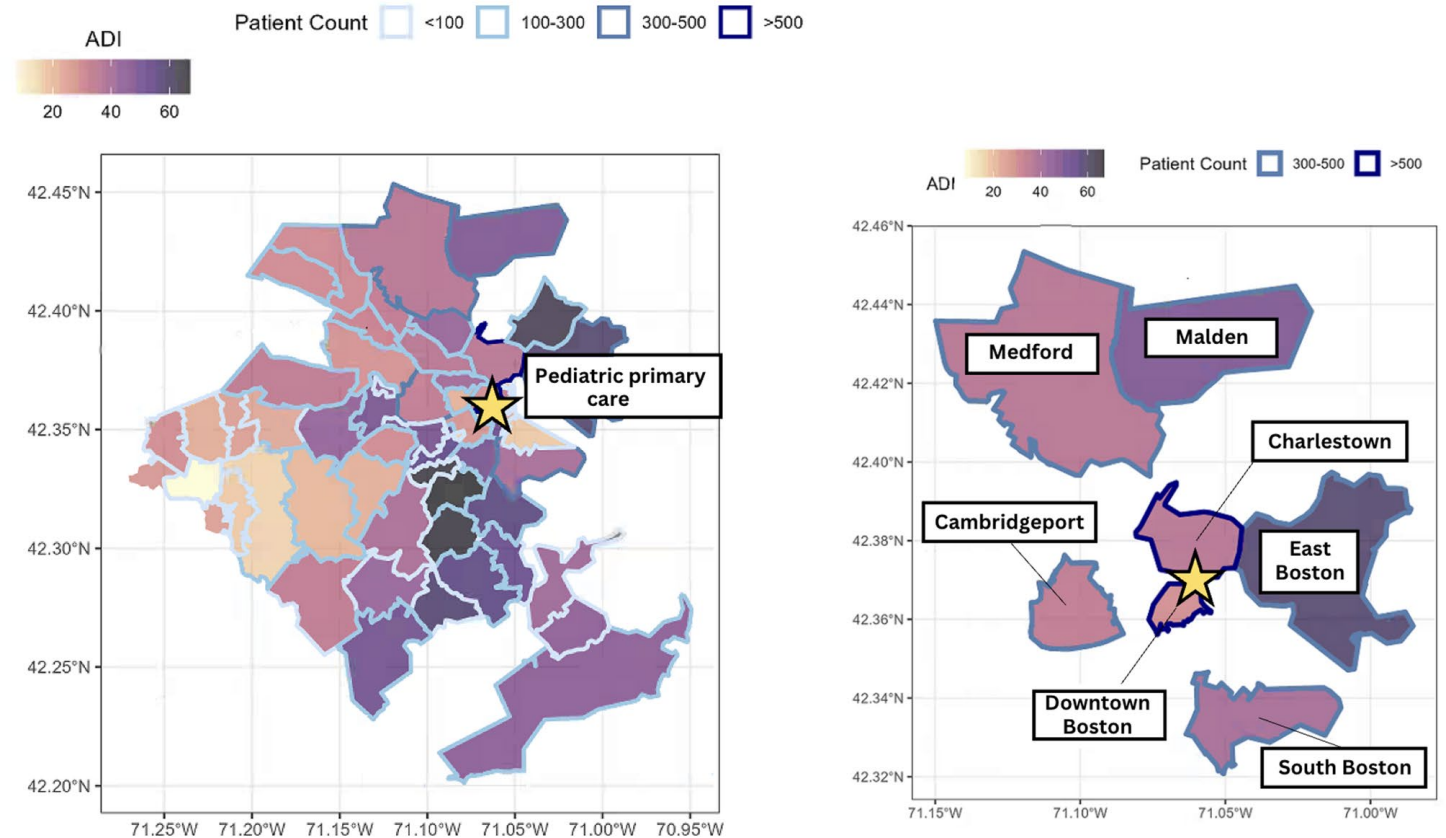
Socioeconomic disadvantage of communities served by pediatric primary care clinics based on patient density. (a) Area deprivation index by zip code. Darker colors indicate greater socioeconomic disadvantage (i.e., poorer access to public goods such as education, transportation, housing, and employment). (b) Area deprivation index (i.e., socioeconomic disadvantage) for neighborhoods with the highest density of patients served by the pediatric primary care clinics

MGH pediatric primary care clinics serve the greater Boston area (see Fig. 4). To better understand the socioeconomic context of patient communities, we examined census-level data based on patient density and neighborhoods (i.e., zip code) among patients served from July 1, 2023 through June 30, 2024. Specifically, we examined the area deprivation index (ADI) [79, 80], which represents a unified index of social determinants of health, including access to transportation, education, employment, housing, and food [81]. A higher ADI indicates greater socioeconomic disadvantage and lower access to social determinants of health. The patient density for each region is depicted by its border color, with darker borders indicating regions with a higher density of patients served by the pediatric primary care clinics.

Taken together, these figures show that patient communities served by the pediatric primary care clinics vary in socioeconomic disadvantage and social determinants of health. Communities on the western perimeter of the catchment area are relatively well-resourced, whereas disadvantage is higher in the eastern and south-central regions of Greater Boston. The diverse socioeconomic makeup of patients’ neighborhoods underscores the need for routine social determinants of health screening to identify and address resource shortages among families to prevent deleterious health outcomes [82]. Accordingly, our pediatric primary care clinics conduct universal parent and child screenings of developmental, emotional, and mental health (e.g., Patient Health Questionnaire - Adolescents, [83]; Pediatric Symptom Checklist-17 [PSC-17], [84]; Generalized Anxiety Disorder-7 [GAD-7], [85]; Survey of Wellbeing of Young Children, [86]; and social determinants of health using an internal 10-item screener assessing transportation, food, housing, utility, financial, and technology needs; see [87, 88] for similar screeners in pediatric primary care). This screening strategy is a key component of a system of care that is responsive to multilevel trauma and adversity (e.g., socioeconomic disadvantage, lack of access to public goods) that can impact patient health. While these screeners may indicate exposures to adversities (e.g., housing insecurity) or some symptoms commonly seen in the context of trauma exposure but are not unique to experiencing trauma (e.g., sleep problems, negative thoughts and mood) they do not universally assess for PTSD symptoms (e.g., reexperiencing, avoidance) tied to a particular trauma exposure.

Given our local community’s diverse socioeconomic makeup (Fig. 4) and the peri-pandemic surge in youth mental health needs [89], we developed and piloted integrated mental health services in primary care (MGH IPC) and trauma-informed psychotherapies in secondary care as new tiers of support. With these programs in place, pediatricians were able to begin referring pediatric primary care patients to MGH IPC for enhanced screening, interventions, and triage to a range of specialized programs within MGH or the community, including the trauma-informed psychotherapy interventions.

#### II. Programmatic description of the Massachusetts General Hospital Integrated Primary Care (MGH IPC) program

Integrated care in pediatric settings has been used for nearly five decades [51–53, 90]. The level of integration varies by model, ranging from co-location, wherein mental health clinicians practice within primary care clinics, to full integration, in which multidisciplinary team members provide joint, coordinated care in the same clinical space [91]. In Massachusetts, pilot programs have successfully increased primary care providers’ ability to consult with mental health specialists, providing a pathway to expand integrated models of care in our clinic [92, 93]. Established in October 2021, MGH IPC clinicians are embedded within the primary care clinics and integrated into the workflow of the primary care team (e.g., pediatricians, nurses, nutritionists). Akin to a “firehouse” model, wherein staff are on standby in the clinic, MGH IPC clinicians spend half of their working hours in clinic shifts. They participate in a combination of pre-scheduled embedded consults, remain on-call for emergent concerns, or provide “curbside consults” to discuss patient-specific questions with pediatricians as needed (without providing direct care).

MGH IPC services include insurance-billable clinical procedures and non-billable activities critical to caring for children (e.g., record review, coordinating care with schools and medical specialists, triage to tertiary care, curbside consultations to pediatricians). An internal review found that, currently, only 40.4% of our embedded care consults are billable (see Supplementary Fig. S1 [78] for details). MGH psychiatry departmental seed funding and additional philanthropic support have expanded our team to currently cover 60% of our primary care clinics in our downtown Boston location.

### Screening and follow up

Referral to an embedded MGH IPC clinician can be initiated in multiple ways. The MGH IPC program has a “low threshold” for referral to be able to address focal concerns or preventive guidance needs, in addition to more complex mental health concerns (see Fig. 3). The primary care team proactively includes MGH IPC clinicians in appointments with patients who have previously identified complex or acute concerns (e.g., suicidality, substance use, non-suicidal self-injurious behaviors). Patients are also referred to an MGH IPC clinician when their scores on the aforementioned universal screeners indicate need for further screening or intervention (e.g., PSC-17 score greater than 14; GAD-7 score greater than 6). Finally, patients may be referred based on pediatrician, patient, or family reports or for developmental guidance (e.g., toileting, sleep), peer-related difficulties (e.g., bullying), emotion or behavior dysregulation (e.g., aggression, mood changes), or family changes (e.g., conflict). Based on medical record reviews, information from the primary care team, and clinical interviews, MGH IPC clinicians assess a range of developmental, mental health, and safety concerns to develop an individualized follow-up plan in collaboration with the patient, family, and primary care team. 

MGH IPC clinicians provide a range of services in initial and follow up appointments, including warm handoffs (e.g., 10-minute introduction to patient via a member of the primary care team with planned follow up; see [94]), brief assessment with psychoeducation, brief interventions, evaluation and treatment planning, urgent psychopharmacology evaluation, comprehensive evaluations of 60 minutes or longer with triage to acute services (e.g., psychiatric emergency services, intensive outpatient, partial hospitalization programs), or care planning in which clinicians collaborate with patients, their families, and the primary care team to enhance continuity of care. Universal screening for specific safety-related concerns (e.g., postpartum depression, suicidality, domestic violence, homicidality) is conducted by the primary care team and/or MGH IPC clinicians in compliance with national, state, and hospital policies [95, 96]. In addition to screening for such targeted safety-related concerns, IPC clinicians also assess whether there have been significant changes for the child or family, recent stressors or trauma, or if they have any additional safety-related concerns. Pediatricians may also report histories of trauma for children to IPC clinicians based on their past assessments with the family to facilitate patient care. If a child has been exposed to potentially traumatic events, clinicians assess for PTSD symptoms through unstructured clinical interviews based on DSM-5-TR criteria and may include a standardized tool (e.g., Child and Adolescent Trauma Screen, [97]; Child PTSD Symptom Scale for DSM-5, [98]; Young Child PTSD Screen, [99]) if further PTSD screening is warranted. Patients are referred within the MGH hospital system for further evaluations and interventions (e.g., neuropsychological testing, psychotherapy, psychiatry for medication management, interdisciplinary feeding program, obsessive-compulsive disorder clinic, substance use disorders clinic) or to community-based services (e.g., in-home or occupational therapy) when appropriate. If a child is experiencing PTSD symptoms such as re-experiencing, avoidance, and hyperarousal symptoms, they are referred for outpatient trauma-focused therapy (e.g., Trauma-Focused Cognitive Behavioral Therapy [100]; Child-Parent Psychotherapy [101]). A subset of patients with emerging emotion dysregulation problems (e.g., frequent or developmentally inappropriate meltdowns, tantrums, inability to self-soothe, hitting) are referred to our family-centered, trauma-informed psychotherapy interventions within our third tier of care (see Fig. 3), described in the following section. 

#### II. Programmatic description of MGH trauma-informed psychotherapies

Established in July 2023 with two clinical psychologists within our child psychiatry outpatient clinic, the third tier of our model of care consists of two trauma-informed psychoeducational and skills-focused group-based psychotherapy interventions for pre-adolescent children (ages 2–12) and their parents. MGH IPC patients who present with emotion or behavior dysregulation and who do not have existing mental health care are eligible. Emotion dysregulation is a transdiagnostic process commonly associated with trauma and adversity [68–73, 102], emerging behavioral difficulties [73, 76], and risk for future psychopathology [71, 73–75] that can be effectively targeted in group-based interventions (e.g [103, 104]). Based on this evidence, we designed these interventions using a transdiagnostic approach, such that children with a variety of diagnoses (e.g., ADHD, adjustment disorder, behavioral concerns) are eligible, so long as their primary presenting concern is emotion dysregulation (see Supplemental Table S1).

Children referred to these interventions complete an initial 60-minute evaluation with a clinician, including the child’s developmental, medical, and mental health history and a thorough safety assessment (including history of trauma or adversity). The intake process for both trauma-informed interventions include asking families about a range of child and family stressors (e.g., “Have you or your child experienced any changes or stressors recently? Do you feel safe at home? Do you have any concerns about violence in the home? Are there any guns in the home? Do you have any other safety-related concerns? Has your family ever had any involvement with Child Protective Services? Is there anything else that we did not discuss that would be important or helpful for me to know about?”). Similar to IPC appointments, if parents report potentially traumatic events, clinicians assess DSM-5-TR criteria-based PTSD symptoms for the child through clinical interview, assessing for traumas or stressors that may be affecting the child’s current presentation. Thus, we do not screen universally for lifetime exposure to a comprehensive list of Criterion A traumas through standardized screeners. Based on parental report and clinician interview and observations, standardized assessments for DSM-5-TR criteria-based PTSD symptoms for children may be used as a follow up to the initial open-ended clinical interview, as noted earlier. As with IPC visits, if a child is experiencing acute PTSD symptoms, they are referred to outpatient trauma-focused therapy. However, our trauma-informed interventions do include children and families with lifetime histories of a range of adversities or potentially traumatic experiences and emotion dysregulation symptoms (e.g., sleep problems, negative thoughts and mood, irritability) who do not meet current criteria for a PTSD diagnosis.

Rooted in evidence-based interventions that target parent-child relational functioning (e.g [103, 104]), both interventions address emotion regulation to reduce risk for later psychopathologies [105]. By targeting a transdiagnostic mechanism in a group setting, these interventions facilitate timely access to care for a subset of MGH IPC patients with diverse mental health concerns. Given the prevalence of trauma and adversity in the general population [9, 10], and especially among children with emotion dysregulation [68–73, 102], we hypothesized that this subset of patients would be more likely to have experienced trauma or adversity than primary care patients. Accordingly, we integrated a trauma-informed approach on an a priori basis (see Fig. 1 [25] for a description of how interventions integrate SAMHSA’s principles of trauma-informed care).

### Young Child Parenting Group (Ages 2–6)

This 10-week group for parents of children 2–6 years integrates principles from Strong Roots interventions [103] and Parent-Child Interaction Therapy [106]. Core elements of the intervention include: psychoeducation about normative social-emotional development, behavioral skills to effectively target challenging behaviors and increase positive behaviors, and relationally-oriented strategies to improve parents’ capacity to co-regulate with their children. Based on information parents share in the group sessions related to current familial stressors and child behaviors, clinicians provide psychoeducation about how parent-child interactions are often linked to parents’ own childhoods. Interventions aim to empower parents to recognize maladaptive multigenerational cycles, strengthen emotional attunement to their child, reduce their child’s behavioral difficulties, and effectively manage strong emotions for themselves and their children.

Upon the completion of each group cycle, parents participate in an individual wrap-up session to identify ongoing family needs and connect with additional services as needed. Parents also complete anonymous online quality improvement surveys to provide feedback about their experiences. Preliminary results suggest that parents find the group to be acceptable and helpful (See Supplemental Tables S2 and S4).

### Dialectical Behavioral Therapy for Elementary School-Aged Kids (DBT-E; ages 7–12)

DBT-E serves families with children 7–12 years. This intervention is adapted from DBT [107] and DBT for Adolescents [104], and also incorporates parenting strategies from Parent Management Training [108]. This 3-month intervention includes 10 weekly parenting skills group sessions, four parent guidance sessions individualized for each family, and seven family therapy sessions with parents and their child. Ad-hoc parent coaching is available between sessions. Consistent with DBT’s transactional model, DBT-E recognizes the bidirectional impact of interactions between a child and their parents, which create cycles that either reinforce or reduce a child’s emotion dysregulation. By helping parents increase their distress tolerance and emotion regulation skills, they can respond more effectively to their children’s challenging behaviors rather than reacting impulsively. Working with both the child and their parents makes it possible to break negative cycles and foster a more regulated, validating environment that promotes adaptive behaviors. Parents in the DBT-E intervention also complete an anonymous online quality improvement feedback survey during their final session. Results suggest parents find DBT-E to be helpful and acceptable (see Supplemental Tables S3 and S5).

#### Part II: Demographics and lifetime prevalence of trauma and adversity in primary and secondary care clinics

This retrospective study used three data sets from primary and secondary care clinics of a large academic medical center within the Massachusetts General Hospital (MGH) hospital system in Boston, Massachusetts to describe and compare patients served by each level of our tiered model of care during the first year of full implementation. First, we used an institutional data repository that included all patients served in pediatric primary care (infants - age 21 years) in downtown Boston where our programs are housed (*n* = 9535). This large dataset characterizes the full sample of pediatric primary care patients served by the facility during the study period. Second, we used an internal clinical dataset that documents all patients referred by their primary care providers to MGH IPC (*n* = 267). Third, we used an internal clinical dataset that included all patients enrolled in one of our trauma-informed psychotherapy interventions (*n* = 63). These datasets are nested within one another, such that a subset of patients seen in pediatric primary care were referred to MGH IPC for integrated follow-up, and then a subset of MGH IPC patients were referred to trauma-informed psychotherapy for tailored intervention. All datasets were harmonized to cover the same one-year study period from July 1, 2023 to June 30, 2024. All study procedures were reviewed and approved by the Mass General Brigham Institutional Review Board (IRB) (Protocol # 2023P001337). The Mass General Brigham IRB determined that this secondary research study does not require consent and meets the criteria under exemption 45 Common Federal Rule (CFR) § 46.104(d)(#). The Mass General Brigham IRB’s review practices and the present study are in compliance with the Helsinki Declaration [109].

## Procedures

### I. Secondary data analysis of pediatric primary care using an institutional data repository

To provide a broader context for sample estimates of patients from MGH IPC and our trauma-informed psychotherapy interventions, we conducted secondary data analyses using our institution’s Complete Patient Data Science Repository (PDSR) Curated Data Set [110]. The PDSR repository contains over 5 billion clinical observations from the entire Mass General Brigham patient population, including, but not limited to, patient demographics, diagnoses, laboratory tests, medications, procedures, and reasons for visits. All data are standardized using the Observational Medical Outcomes Partnership Common Data Model v5.2 and updated monthly. Mass General Brigham-affiliated researchers can access and conduct analyses without additional IRB approval, as the repository contains limited Protected Health Information (PHI), and identifiers are removed in accordance with institutional policies [110] and the Health Insurance Portability and Accountability Act (HIPPA) Privacy Rule at 45 CFR § 164.514(e)(4) [111]. The Mass General Brigham IRB’s review practices and the present study are in compliance with the Helsinki Declaration [109].

### II. Retrospective review of medical records for MGH IPC and trauma-informed psychotherapy interventions

#### Data collection

Licensed clinical psychologists and supervised psychology postdoctoral fellows recorded each patient encounter within MGH IPC and the trauma-informed psychotherapy interventions between July 1, 2023 and June 30, 2024. In addition to documenting each clinical encounter in the patient’s medical chart within the hospital system’s medical recordkeeping system, per standard documentation and billing procedures, we also maintain a patient log in Excel, which includes medical record numbers, encounter dates, demographics, and clinical information (e.g., presenting concerns, mental health diagnoses) for program development purposes.

#### Data coding

A retrospective review of medical records was conducted for all patients who received services from MGH IPC (*n* = 267) and/or participated in the trauma-informed psychotherapy interventions (*n* = 63) between July 1, 2023 and June 30, 2024. Data coders individually reviewed each participant’s medical chart to code for the following variables: age, sex, race, ethnicity, insurance, billing diagnosis, and lifetime trauma and adversity. Patient demographics were extracted from pre-existing fields in the medical records.

#### Lifetime prevalence of trauma and adversity

We reviewed visit notes in the patient’s chart to code lifetime potentially traumatic experiences as defined by the DSM-5-TR’s Criterion A [17] and a range of adversities. All available clinical documentation from all providers in the chart was included in our search. We developed a list of 19 adverse and potentially traumatic experiences based on the original 10-item Adverse Childhood Experiences Questionnaire (ACEs Questionnaire, [4]), the 17-item Pediatric Adverse Childhood Experiences and Related Life Events Screener (PEARLS, [112]), and the 15-item Philadelphia Adverse Childhood Experiences Survey (PHL ACEs, [16]). The PEARLS and PHL ACEs both adapt and expand the original ACEs Questionnaire to include additional adversities and traumas such as parent physical illness/disability, housing instability, poverty, bullying, foster care, discrimination, and community violence to improve population-level surveillance. We combined the originally separate ACEs items regarding parent substance use and parent mental illness into one item, “parent mental health/trauma history.” We included all adversities and traumas that appeared in any of the three questionnaires to develop a comprehensive list of 19 adversities and traumas. See Table 1 [4, 16, 112] for the full list of items. We categorized these traumas and adversities into three socioecological domains: “Child” (e.g., child abuse/neglect), “Family Conflict/Instability” (e.g., parent incarceration or mental illness), and “Community Violence/Neighborhood Safety” (e.g., witnessing neighborhood violence). We coded potentially traumatic experiences not specified in the ACEs Questionnaires (e.g., natural disaster, motor vehicle accident) as “Other trauma” under the “Child” domain. To calculate the percentage of participants who experienced at least one adverse or traumatic experience in their lifetime, we used our comprehensive 19-item list of adverse and traumatic experiences. Additionally, to align with existing epidemiological research (see [113] for review), we calculated a separate percentage of participants who experienced at least one of the original 10 items on the ACEs Questionnaire [4]. We report prevalence rates for both the original ACEs Questionnaire items and the comprehensive list of 19 adverse and traumatic experiences in Table 1 [4, 16, 112].

**Table 1:** Prevalence of trauma and adversities for patients in MGH IPC and trauma-informed psychotherapy interventions

Adversity level	Item	Prevalence (%)		Chi-square
		**MGH IPC** **(n = 267)**	**Trauma-informed psychotherapy** **(n = 63)**	**χ**^**2**^**(*df*** **= 1)** ***p***
Child	Child abuse – physical*	15 (5.6%)	-	
	Child abuse – sexual*	-	-	
	Child abuse – emotional or verbal*	11 (4.1%)	-	
	Child neglect*	15 (5.6%)	-	
	Child prejudice or discrimination	-	-	
	Child medical trauma	156 (58.4%)	34 (54.0%)	
	Bereavement	20 (7.5%)	-	
	DCFS involvement – current	18 (6.7%)	-	
	DCFS involvement – prior	57 (21.3%)	11 (17.5%)	
	DCFS involvement – placement*	-	-	
	Other trauma	80 (30.0%)	20 (31.7%)	
	*At least 1 child item*	198 (74.2%)	42 (66.7%)	
Family	Parent domestic violence*	50 (18.7%)	11 (17.5%)	
	Parent incarceration*	10 (3.7%)	-	
	Parent mental health/trauma history*	171 (64.0%)	60 (95.2%)	
	Parent separation/divorce*	106 (39.7%)	12 (19.0%)	
	Parent significant medical/physical illness	49 (18.4%)	18 (28.6%)	
	Family economic stress	75 (28.1%)	24 (38.1%)	
	Family history of housing instability	36 (13.5%)	13 (20.6%)	
	*At least 1 family item*	226 (84.6%)	62 (98.4%)	
Community	Community violence/neighborhood safety	-	-	
	*At least 1 community item*	-	-	
Overall	*At least 1 item (original ACEs * *Questionnaire)*	205 (76.8%)	62 (98.4%)	*14.08* ^†^ *< 0.001*
	*At least 1 item (19-item expanded list)*	252 (94.4%)	62 (98.4%)	1.03 *0.311*

* Designates items included in the Adverse Childhood Experiences Questionnaire (ACEs Questionnaire, [4]). Comprehensive ACEs list includes the ACEs Questionnaire and additional items from the Pediatric ACEs and Related Life-events Screener (PEARLS, Ye et al. [112]) and Philadelphia Adverse Childhood Experiences (PHL ACEs, Cronholm et al. [16]). DCFS is the abbreviation of Department of Child and Family Services. - Denotes a frequency (#) of less than 10. ^†^ Denotes significant difference between MGH IPC and trauma-informed psychotherapy interventions based on chi-square test of difference

To ensure consistent and reliable coding procedures across the three coders, 25% of the total patient records were double-coded independently (*n* = 85). Inter-rater reliability, calculated using Cohen’s kappa, was high for all trauma/adversity categories, including child-level adversities (α_child_ = 0.95), family-level adversities (α_family_ = 0.89), community-level adversities (α_community_ = 1.0), and an overall category capturing exposure to any of the 19 traumas or adversities (α_overall_ = 0.82).

#### Data analyses

All data cleaning and analyses were performed in R version 4.4.1 [114]. A two-tailed t-test at a significance level of 0.025 was used to compare the mean age of the pediatric primary care population and MGH IPC patients. Chi-square tests were conducted to compare patient sex, race, and ethnicity within the pediatric primary care population, MGH IPC, and the trauma-informed psychotherapy interventions. Chi-square tests were also used to compare sex and insurance status between patients in MGH IPC and patients in the trauma-informed psychotherapy interventions. We were unable to conduct comparisons of insurance status for the pediatric primary care population, as insurance status was not available for the pediatric primary care clinics in the institutional data repository.

## Results

### Study sample

#### I. Demographics

Demographic information for patients in all three care settings (pediatric primary care, MGH IPC program, and trauma-informed psychotherapy interventions) is reported in Table 2. Overall, the patients referred to MGH IPC were similar to the pediatric primary care population in both age and sex at birth. Children in the trauma-informed psychotherapy interventions were expectedly younger (*M* = 5.9, SD = 2.9) than children in pediatric primary care (*M* = 9.6, SD = 6.2) and MGH IPC (*M* = 10.2, SD = 5.5), which is consistent with the different age ranges served by the respective interventions. There was a significant difference in the racial makeup of the MGH IPC program and pediatric primary care samples, where a larger proportion of patients in IPC identified as White compared to the pediatric primary care population (59.6% vs. 49.8%, χ^2^ = 10.88, *p* =.012). The trauma-informed psychotherapy interventions also had a higher proportion of patients who identified as White compared to pediatric primary care (63.5% vs. 49.8%, χ^2^ = 4.14, *p* =.042). There were no significant differences in sex or insurance status between patients in MGH IPC and the trauma-informed psychotherapy interventions. See Tables 3, 4 and 5 for chi-square comparisons of demographics across care settings.

**Table 2:** Demographics of patients in pediatric primary care, MGH IPC, and trauma-informed psychotherapy interventions

	Pediatric primary care	MGH IPC	Trauma-informed psychotherapy
Sample size (n)	9535	267	63
Age (mean (SD))	9.6 (6.2)	10.2 (5.5)	5.9 (2.9)
Sex at birth (n %)			
Female	4841 (50.8%)	136 (50.9%)	33 (52.4%)
Male	4694 (49.2%)	131 (49.1%)	30 (47.6%)
Race (%)			
Asian	934 (9.8%)	16 (6.0%)	-
Black	1172 (12.3%)	38 (14.2%)	-
Other	1614 (16.9%)	35 (13.1%)	-
White	4751 (49.8%)	159 (59.6%)	40 (63.5%)
Unavailable	1064 (11.2%)	19 (7.1%)	-
Ethnicity (%)			
Hispanic	1266 (13.3%)	43 (16.1%)	-
Non-Hispanic	6756 (70.9%)	198 (74.2%)	42 (66.7%)
Unavailable	1513 (15.9%)	26 (9.7%)	12 (19.0%)
Public insurance (%)	n/a	67 (25.1%)	15 (23.8%)

- Denotes a frequency (#) of less than 10. n/a denotes the variable was not available in the dataset

**Table 3:** Chi-square comparisons of demographics between pediatric primary care and MGH IPC

Variable	Pediatric primary care	MGH IPC	Chi-square test for independence
Sex			χ2 *(x) = 3.03e-28*
Male Female	4694 (49.2%) 4841(50.8%)	131 (49.1%) 136 (50.9%)	*p* = 1.000 *df = 1*
Race			
Asian Black Other White	934 (9.8%) 1172 (12.3%) 1614 (16.9%) 4751 (49.8%)	16 (6.0%) 38 (14.2%) 35 (13.1%) 159 (59.6%)	χ2 *(x) = 10.88* *p* =.012 *df = 3*
Ethnicity			χ2 *(x) = 0.60*
Hispanic Non-Hispanic	1266 (13.3%) 6756 (70.9%)	43 (16.1%) 198 (74.2%)	*p* =.439 *df = 1*

**Table 4:** Chi-square comparisons of demographics between pediatric primary care and trauma-informed psychotherapy interventions

Variable	Pediatric primary care	Trauma-informed psychotherapy	Chi-square test for independence
Sex			χ2 *(x) = 0.02*
Male Female	4694 (49.2%) 4841(50.8%)	30 (47.6%) 33 (52.4%)	*p* =.898 *df = 1*
Race			χ2 *(x) = 4.14*
White Non-White	4751 (49.8%) 4784 (50.2%)	40 (63.5%) 23 (36.5%)	*p* =.042 *df = 1*

**Table 5:** Chi-square comparisons of demographics between MGH IPC and trauma-informed psychotherapy interventions

Variable	MGH IPC	Trauma-informed psychotherapy	Chi-square test of independence
Sex			χ2 *(x) =* 0.004
Male Female	131 (49.1%) 136 (50.9%)	30 (47.6%) 33 (52.4%)	*p =*.947 df = 1
Race			χ2 *(x) =* 0.187
White Non-White	159 (59.6%) 108 (40.4)	40 (63.5%) 23 (36.5%)	*p =*.666 df = 1
Public insurance			χ2 *(x) =* 0.003
Yes No	67 (25.1%) 200 (74.9%)	15 (23.8%) 48 (76.2%)	*p =*.96 df = 1

#### II. Lifetime histories of trauma and adversity

The prevalence of patients’ lifetime exposure to adverse and/or potentially traumatic events are presented in Table 1 [4, 16, 112]. These are categorized by the socioecological level in which the trauma or adversity occurred, with traumas or adversities directly impacting the child categorized under the “Child” level, trauma or adversities occurring within the family system categorized under the “Family” level, and community violence or neighborhood-based traumas or adversities categorized under the “Community” level. Based on our comprehensive 19-item list of traumas and adversities, 94.4% of patients in MGH IPC and 98.4% of children in the trauma-informed psychotherapy programs had evidence of one or more such experiences in their lifetime. These overall prevalence rates were not statistically different (χ^2^ = 1.03, *p* =.311). Over half of the children in both MGH IPC and trauma-informed psychotherapies had a history of medical trauma, which may be related to the fact that we are located in a large urban general hospital that is equipped to treat a higher volume of complex patients compared to community-based clinics. Lifetime prevalence of a family-level trauma or adversity was highest among children in the trauma-informed psychotherapies, where 95.2% had a parent with a significant mental health concern or trauma history. Roughly one-third of all patients had a documented “other trauma,” defined as a potentially traumatic event that did not fall under one of the other adversity categories (e.g., suicide attempt of a loved one). When limiting the list of adverse experiences to the original 10 items in the ACEs Questionnaire [4], we saw significant differences between MGH IPC and trauma-informed psychotherapies, where 76.8% of MGH IPC patients and 98.4% of trauma-informed psychotherapy patients had a lifetime history of at least one ACE (χ^2^ = 14.08, *p* = < 0.001), suggesting that the population served by the trauma-informed psychotherapy interventions includes families with more child and family-level adversities

## Discussion

This study describes the conceptual basis and pilot implementation of a tiered model of pediatric mental health care in a large academic medical center. Descriptive data from a retrospective record review of the lifetime prevalence of adversity and trauma provides strong evidence in support of implementing trauma-informed population mental health approaches in pediatric healthcare. First, we discuss the implications of our findings for clinical frameworks, focusing on the need for developmentally tailored, transdiagnostic, trauma-informed, family-centered care and screening methods. Next, we describe key considerations for policymakers to facilitate equitable capacity building and implementation of trauma-informed care. Finally, we outline future research directions for evaluating the implementation of our model of care to ensure it is effective, financially sustainable, and equitable.

### Implications for clinical frameworks

Our findings support three key considerations for advancing trauma-informed care: (1) the high prevalence of trauma and adversity in pediatric populations warrants a trauma-informed approach to population mental health, (2) family-centered screening and care is central to pediatric mental health, and (3) screening for developmentally salient, transdiagnostic emotion dysregulation symptoms may be a clinically useful method to identify patients in need of further trauma-informed evaluation and intervention.

First, our retrospective record review found that a majority of patients in both our integrated primary care program (MGH IPC) and trauma-informed psychotherapy interventions in a secondary care setting reported experiencing trauma or adversity. Differences emerged depending on how these experiences were operationalized. When we assessed a comprehensive 19-item list of adverse and traumatic experiences based on standardized questionnaires incorporating ACEs [4], PEARLS [112], PHL ACEs [16], and Criterion A traumas [17], the difference in the lifetime prevalence of trauma and adversity between MGH IPC and trauma-informed psychotherapy patients (94.4% vs. 98.4%) was not significant. However, when the prevalence of only the 10 items from the original ACEs study [4] were considered, there was a significant difference in the prevalence between MGH IPC (76.8%) and the trauma-informed psychotherapy interventions (98.4%). The high prevalence of adversity and trauma in these patient populations across both operationalizations of adversity and trauma is notable given that our clinicians do not conduct universal trauma and adversity screenings based on standardized measures.

Second, our findings also support family-centered approaches to trauma-informed screening and care for pediatric patients. Family-level stressors were highly prevalent among our MGH IPC (84.6%) and trauma-informed psychotherapy (98.4%) patients. Parent-related stressors, including parents’ lifetime history of trauma or a significant mental health concern, were prevalent in MGH IPC (64.0%) and the trauma-informed psychotherapy interventions (95.2%), reflecting statewide trends–about 62% of Massachusetts adults report at least one childhood adversity and 16.2% report four or more [115]. Such data are highly relevant to informing clinical frameworks and programming efforts in pediatric mental health care because parents’ trauma and adversity histories and mental health are associated with their own emotion regulation, parent-child interactions, and children’s wellbeing and mental health [116]. Together, this evidence underscores the importance of a family-centered approach in trauma-informed screening and care for children.

Third, our findings suggest that a transdiagnostic, symptom-driven screening approach focused on trauma and adversity-related sequelae (i.e., emotion dysregulation) may be an effective strategy to facilitate early identification in pediatric primary care and follow up in trauma-informed psychotherapy. Evidence regarding the usefulness of universal trauma and adversity screening is mixed [61, 62]. However, even though we did not explicitly screen for a comprehensive list of lifetime DSM-5-TR-based Criterion A traumas or PTSD symptoms, our retrospective record review still found a high prevalence of adversities and trauma, especially parent and family-level adversities, among families referred to the trauma-informed psychotherapies. Future research on factors driving this observation are needed (e.g., our interventions may include parents who were more motivated to participate in treatment due to their mental health or trauma histories). That said, a symptom-driven approach focused on changes in emotional and behavioral functioning may identify children most in need of trauma-informed care. Relatedly, our approach is also aligned with efforts to understand transdiagnostic sequelae of adversity and trauma [1, 3, 102], characterize mental health using cross-cutting measures [117, 118] rather than traditional nosological systems, and shifts to understand common mechanisms of change in psychotherapy interventions [119, 120].

Our findings must be interpreted within the context of some methodological limitations. Our study’s retrospective review of patient medical charts leverages clinically rich assessments made by multidisciplinary healthcare teams, but is likely to result in underreporting. Healthcare visit appointments outside our hospital system may not always be available in patient charts, and documentation may be limited by reporting issues described elsewhere (e.g., delayed or underreporting of trauma and adversity; [121–124]). Systematic screening of adversities and trauma is likely to yield different estimates, particularly for experiences that might be considered distal (e.g., community-based stressors) or less exigent from a safety or medical perspective (e.g., experiences of discrimination). Validation studies comparing retrospective review of patient medical charts data and screening questionnaires and clinician evaluations are necessary next steps. Thus, the current study’s procedures likely underestimate trauma and adversity relative to other methods. It is also important to acknowledge the limitations of the ACEs framework. The ACEs and expanded PEARLs questionnaires were developed for population-level surveillance, rather than individual-level clinical decision-making [4, 63, 112]. Thus, the impact of trauma and adversity, rather than the mere presence or exposure of trauma or adversity, may better inform individual clinical decision making. Additionally, this study could not compare socioeconomic indicators across levels of care (e.g., insurance) because such data were unavailable from our institutional dataset on pediatric primary care clinics. Comparisons of private versus public insurance types between MGH IPC and trauma-informed psychotherapy programs found no significant differences. Relatedly, the limited racial diversity in our trauma-informed psychotherapy groups warrants further research. Although these findings could be due to our small sample size, they may also be attributable to barriers to care access and engagement, inequities in referrals, and provider bias or insufficient cross-cultural competency that disproportionately affect patients of marginalized racial and ethnic identities [125–127]. As such, future assessments with comprehensive socioeconomic and social determinants of health indicators as well as mixed-methods and community participatory studies of barriers to accessing care are necessary to facilitate equitable implementation.

### Considerations for policymakers

While evidence-based capacity development and program implementation are essential, they are insufficient to mitigate the effects of childhood trauma and adversity absent policy reform [26, 128–130]. Health policy is a key pillar of population mental health [131]. Long-standing policy efforts such as the *Paul Wellstone and Pete Domenici Mental Health Parity and Addiction Equity Act* (2008) [132], which seeks to provide equal coverage for physical and mental health services, the Affordable Care Act, which mandates coverage of preventive mental health care for children and postpartum mothers [133], and more recent legislative efforts (i.e., *Investing in Kids’ Mental Health Now Act of 2022* [134]; *Helping Kids Cope Act* [135]) could support programs like ours [52]. However, state-level variability in policy implementation and the changing policy landscape present challenges in leveraging such measures to scale trauma-informed care. A comprehensive review of pediatric mental healthcare policy is outside the scope of our article (see [58, 128–130, 136, 137] for relevant reviews), but we focus on five key considerations for policymakers to facilitate scaling programs like ours.

First, the near-universal prevalence of trauma and adversity, as well as the significant prevalence of economic stress and housing instability in our clinical populations, underscore the need for prevention and cross-sector approaches. Policies that support social determinants of health, such as families’ basic needs and economic security, are crucial to evaluate and expand [138], as such measures have been shown to reduce the overall burden of adversity and improve mental health outcomes [139–143].

Second, scaling trauma-informed models of care requires a national shift in healthcare training to build and sustain workforce capacity. The long-standing shortage of pediatric mental health specialists was exacerbated by the COVID-19 pandemic [39, 144, 145]. Moreover, current training and competency requirements omit trauma curricula. For instance, only 8% of clinical psychology doctoral programs require coursework in trauma-informed care [146], and such curricula are largely absent in other pediatric specialty training [30]. National capacity to provide trauma-informed care is even more constrained when provider diversity is considered [147]. Finally, workforce retention is a key challenge, as high turnover is common among clinicians whose caseloads include a high trauma burden [148, 149]. In accordance with SAMHSA’s principles, attending to provider well-being and offering trauma-informed supervision are essential to building and maintaining trauma-informed systems of care [25], an observation supported by research regarding the risk of secondary traumatization among mental health providers [150].

Third, policy reform is essential to facilitate the delivery of trauma-informed pediatric mental health care. Interventions for children are necessarily family-centered and need to be tailored across a range of developmental stages. Key components of pediatric mental health care, including prevention, early intervention, and psychotherapy interventions (including group-based interventions for parents like ours), are inherently more time-intensive than adult or pharmacological interventions. In part, this is because such care requires consistent coordination with parents, schools, or other providers and necessitates a trust-building, collaborative approach. Current reimbursement policies do not account for this type of care coordination and are limited for services that do not involve direct patient contact. Additionally, as one example specific to our group-based interventions, Current Procedural Terminology (CPT) [151] codes 96202 and 96203 for group psychotherapy with parent(s) (without the pediatric patient present) were only approved in January 2023 but are still not consistently accepted for reimbursement by insurance companies. Consequently, billing under parents’ rather than children’s medical records adds barriers to families’ treatment engagement (e.g., non-billable communications with parents about insurance restrictions, addressing concerns about documentation in parents’ chart, obtaining consent to bill under the parents’ chart).

Fourth, permanent telehealth parity can improve the accessibility of interventions like ours, although ongoing research and adaptations are needed to ensure equity across populations. In Massachusetts, telehealth was expanded under such legislative action [152] during the COVID-19 pandemic, making our interventions feasible. Indeed, our own team’s prior efforts to implement in-person group-based interventions for families were unsuccessful due to a range of barriers for parents (e.g., difficulties in attending multi-week sessions during working hours, transportation, and childcare needs). Feedback from parents helped identify lunch-hour telehealth sessions as the most viable attendance option. Though less suitable in some situations (e.g., patients with safety concerns), telehealth is effective and widely accepted for the treatment of many conditions [153–155], and parity between in-person and remote appointments has been shown to improve access and scalability [156].

Finally, while efforts to integrate mental health care in pediatric primary care have gained momentum [58, 92, 93, 157], such care is still not universally available. In Massachusetts, recent billing models, such as annual mental health screening as part of well-child visits [158], and preventive billing, wherein billable mental health care can be provided without a diagnosis [159], show promise for advancing population mental health. Similarly, enhanced reimbursement and value-based payment models may incentivize prevention and support complex models of care [160, 161]. Finally, newer adaptations of mental health interventions, such as digital mental health tools, can be leveraged in primary care and beyond, but sustainable payment models have yet to be implemented.

### Future directions for implementation evaluation research

Building on the initial success of piloting our tiered model of pediatric mental health care, we identified key implementation research priorities to evaluate effectiveness, fiscal sustainability, and equity and to support efforts to scale our approach.

#### Effectiveness

Both our trauma-informed psychotherapy interventions build on a robust evidence base [103, 104, 106–108]. Additionally, while our quality improvement surveys suggest that parents report finding the interventions acceptable and helpful, assessing the effectiveness of our interventions through pre- and post-intervention multi-method assessments, including a core focus on assessing emotion dysregulation, broadband measures of child behavior functioning (e.g., Child Behavior Checklist, [162]; Difficulties in Emotion Regulation Scale, [163]), and parents (Parental Stress Scale, [164]; Parenting Sense of Competence scale, [165]), are necessary next steps.

#### Fiscal sustainability

Effective scaling necessitates a comprehensive estimation of implementation costs to create sustainable funding mechanisms. As previously noted, integrated mental health in primary care includes a range of non-billable services that are the standard of care for pediatric and trauma-informed care. An internal review of our embedded care consults found that only 40.4% of our embedded consults in MGH IPC are billable and, hence, the majority of visits are sustained by substantial philanthropic support (See Supplemental Fig. S1). With respect to trauma-informed interventions such as ours, a recent review examining 12 evidence-based therapies for children and youth for state-wide scaling identified expected costs–such as licensing, training, and certification–and hidden costs, such as supplies and lost revenue for non-billable services (e.g., care coordination, record review, trauma-informed supervision or case consultation), as key considerations for implementation and sustainability [160]. Using a similar fiscal analysis tool as Hoagwood and colleagues [160], we are currently quantifying the true full cost of implementing our model to ensure financial feasibility, support scaling, and inform policy recommendations to expand sustainable trauma-informed care.

#### Equity

Equitable scaling of trauma-informed care requires ongoing implementation research that considers gaps in access, diverse stakeholder perspectives, and effectiveness evaluations in local communities (see [33]). Our pilot implementation supported equity by prioritizing families without existing mental health care and adapting our intervention to be accessible via telehealth. Additionally, to the best of our knowledge, our program is also the only fully insurance-based pediatric DBT program in Greater Boston. In the next phase, we will pilot a Spanish language adaptation of our Young Child Parenting Group intervention to further increase the accessibility of our programs. Importantly, this analysis found that more patients in the pilot implementation of the MGH IPC and trauma-informed psychotherapy programs identified as White compared to the pediatric primary care population. While differences in racial composition may reflect skewness due to the small sample sizes in this pilot phase, ongoing review of patient demographic composition at each stage of the referral and screening will be critical to ensure equity.

To continue leveraging equity-focused methodologies, we plan to prioritize participatory approaches to clinical program development (e.g., patient advisory boards [166]) and research methods [65, 167, 168], which share principles of trauma-informed care [25, 29]. Such methodologies focus on collaborative partnerships with key stakeholders for whom clinical services are intended or with those who are directly affected by the research (i.e., co-creating research questions, discussing study methodology and results, contextualizing findings within lived experiences, collaborating in dissemination). Clinically, our quality improvement parent surveys are consistent with these approaches. With respect to research, we plan to expand engagement with patients and their families throughout the research process by developing a parent advisory board that will collaborate with our team to plan intervention effectiveness evaluations. 

## Conclusion

Preventing and addressing childhood adversity and trauma are foundational to promoting pediatric population mental health. Our study supports the integration of trauma-informed, transdiagnostic, family-centered screening methods and interventions in pediatric primary and secondary care settings. Findings from our pilot implementation of our tiered model of care, including integrated primary care and trauma-informed psychotherapy programs, underscore the high prevalence of trauma and adversity among children and families. Screening strategies that account for transdiagnostic symptoms like emotion dysregulation and multilevel social determinants of health may improve early identification and intervention. Integrated models of pediatric primary care and group-based, transdiagnostic hybrid interventions like ours can increase accessibility to equitably address the mental health needs of children. Equally important, pediatric trauma-informed care should be family-centered, given the role of the caregiving environment on child development and treatment engagement. Expanding such trauma-informed care will require national shifts in workforce training. Additionally, policies that prioritize cross-sector approaches to address economic insecurity and material needs, and policy changes to promote the fiscal sustainability of evidence-based, developmentally tailored interventions are critical. Finally, equity-focused implementation research and participatory methodologies that facilitate deep engagement with patients and their communities are critical to evaluating intervention effectiveness, scaling delivery models, and, ultimately, improving access to trauma-informed care for children and families.

## Supplementary Information

Supplementary Material 1. [169–171].

## Data Availability

The datasets generated and/or analyzed during the current study are not publicly available due to patient privacy restrictions. All data generated for the purposes of this manuscript are included in this published article.

## Abbreviations

ACE: Adverse childhood experience
ADHD: Attention deficit hyperactivity disorder
ASD: Autism spectrum disorder
CFR: Common Federal Rule
CPT: Current Procedural Terminology
Criterion A trauma: According to the DSM-5-TR, exposure to a Criterion A trauma, which is defined as “actual or threatened death, serious injury, or sexual violence”, is a necessary criterion for diagnosis of posttraumatic stress disorder (PTSD) [17]
DBT: Dialectical Behavioral Therapy
DBT-E: Dialectical Behavioral Therapy for Elementary School-Aged Kids
DCFS: Department of Child and Family Services
DSM-5-TR: Published by the American Psychiatric Association, the *Diagnostic and Statistical Manual of Mental Disorders, Fifth Edition, Text Revision* [17] is a standardized classification system of mental disorders widely used by mental health clinicians and researchers in the United States. It provides a set of diagnostic criteria for more than 70 diagnoses
GAD-7: Generalized Anxiety Disorder 7-item scale
HIPPA: Health Insurance Portability and Accountability Act
IRB: Institutional Review Board
MGH: Massachusetts General Hospital
MGH IPC: Massachusetts General Hospital Integrated Primary Care
PDSR: Patient Data Science Repository Curated Data Set
PEARLS: Pediatric Adverse Childhood Experiences and Related Life Events Screener
PHI: Protected Health Information
PHL ACEs: Philadelphia Adverse Childhood Experiences
PSC-17: Pediatric Symptom Checklist-17
PTSD: Posttraumatic stress disorder
SAMHSA: Substance Abuse and Mental Health Services Administration
US: United States
USA: United States of America
