# Supplementary material for: Implementing a trauma-informed approach in a tiered model of pediatric population mental health care: a pilot study in primary and secondary care
Source: BMC Health Serv Res. 2025 Dec 18;25:1603. doi: 10.1186/s12913-025-13356-7 (PMC12715910; doi:10.1186/s12913-025-13356-7)
Supplement: Supplementary file 1 — Supplementary Material 1. [169–171]. [file 12913_2025_13356_MOESM1_ESM.docx]

**Supplemental Materials**

###### **S1: Diagnostic impressions for pediatric primary care, MGH IPC, and trauma-informed psychotherapy interventions**

*Coding procedures*

To code diagnostic impressions, data coders reviewed patients’ medical records for billing diagnoses during encounters with MGH IPC and/or trauma-informed psychotherapy providers. Because 59.6% of MGH IPC encounters are not billable (see above), when a billing diagnosis was not available for an MGH IPC encounter, we used the pediatrician’s billing diagnosis for the same episode of care. For example, if a child saw their pediatrician and the pediatrician brought in an MGH IPC provider during the visit for a brief introduction (not a billable encounter for MGH IPC), coders used the billing diagnosis for the child’s visit with their pediatrician which prompted the MGH IPC referral. Some MGH IPC patients (13.9%) did not have a mental health-related billing diagnosis from MGH IPC or from their pediatrician. In these cases, their visit with an MGH IPC provider was not billed, and their pediatrician’s billing diagnoses did not include mental health-related diagnoses. The research team consulted the phenome-wide association studies (PheWAS) catalog [169] to identify clusters of diagnostic codes (i.e., phecodes) related to mental health concerns [170, 171]. We used these phecodes to develop a list of mental health codes to categorize patients’ diagnostic impressions. For example, ADHD has multiple subtypes that each have a unique diagnostic code. We coded all ADHD-related diagnoses under a single “ADHD” code. Similarly, anxiety-related disorders (e.g., generalized anxiety disorder, separation anxiety) were coded as “Anxiety.” Infrequent diagnoses were combined into an “Other” category. Diagnosis prevalence rates were calculated for each sample based on the percentage of patients in each dataset who received the diagnosis during the study period.


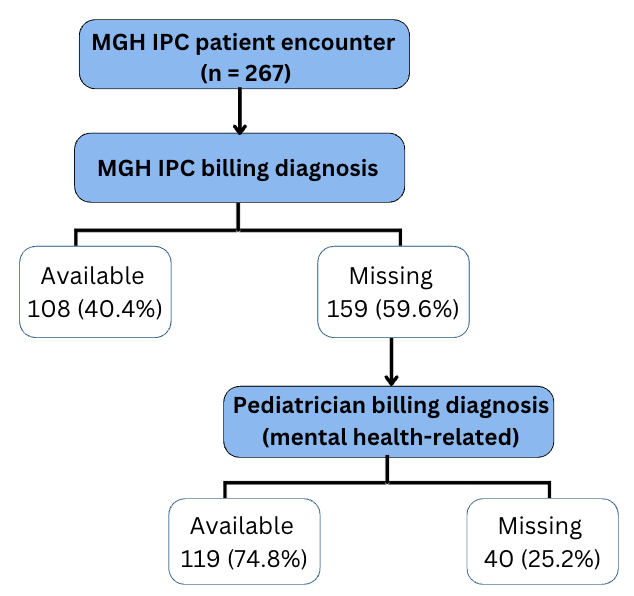


**Fig. S1** Availability of mental health-related billing diagnoses within MGH IPC program and pediatrician visits

*Results*

Anxiety and ADHD were among the most common billing diagnoses across all settings. Among the 9,535 patients seen in pediatric primary care during the study year, 18.6% had at least one mental health-related billing diagnosis. The most common mental health billing diagnoses within pediatric primary care were anxiety (4.6%), ADHD (4.3%), autism (1.8%), eating disorder (1.5%), and speech and language disorder (1.4%). Within MGH IPC, the most common billing diagnoses were anxiety disorders, ADHD, no diagnosis, other, and depressive disorders. Interestingly, depressive disorders were a top diagnosis among children referred to MGH IPC (10.9%), but these were not among the most common diagnoses in pediatric primary care or in the trauma-informed psychotherapy interventions. Adjustment disorder, which is a common diagnosis given to children with emerging or subthreshold mental health concerns, appeared as a top diagnosis only within the trauma-informed psychotherapy interventions (47.6%). This was primarily driven by the Young Child Parenting Group, where 68.3% of children received a diagnosis of adjustment disorder. These younger children typically present with emerging concerns and are less likely to meet the criteria for a specific diagnosis. In contrast, the most prevalent diagnoses among DBT-E patients were anxiety (81.8%) and ADHD (59.1%). Only 9.1% of DBT-E patients were diagnosed with adjustment disorder. Infrequent diagnoses were categorized under “Other,” and this “Other” diagnostic category was the fourth most common diagnosis within both MGH IPC and the trauma-informed psychotherapy interventions, highlighting the wide range of presentations that are referred for further mental health support in MGH IPC.

**Table S1** Most prevalent billing diagnoses in pediatric primary care, MGH IPC, and trauma-informed psychotherapy interventions

| **Pediatric primary care**  **(n = 9535)** | | **MGH IPC**  **(n = 267)** | | **Trauma-informed psychotherapy**  **(n = 63)** | |
| --- | --- | --- | --- | --- | --- |
| **Diagnosis** | **Frequency (%)** | **Diagnosis** | **Frequency (%)** | **Diagnosis** | **Frequency (%)** |
| 1. Anxiety Disorder | 434 (4.6%) | 1. Anxiety Disorder | 86 (32.2%) | 1. Adjustment Disorder | 30 (47.6%) |
| 2. ADHD | 407 (4.3%) | 2. ADHD | 39 (14.6%) | 2. Anxiety Disorder | 22 (34.9%) |
| 3. Autism Spectrum Disorder | 169 (1.8%) | 3. Other | 34 (12.7%) | 3. ADHD | 14 (22.2%) |
| 4. Eating Disorder | 146 (1.5%) | 4. Depressive Disorder | 29 (10.9%) | 4. Other | - |
| 5. Speech and Language Disorder | 138 (1.4%) | 5. Adjustment Disorder | 25 (9.4%) | 5. Depressive Disorder*  5. Autism Spectrum Disorder* | -  - |

Note. * Denotes diagnoses with equal frequencies. - Denotes a frequency (#) of less than 10. Among MGH IPC patients, 59.6% did not have a billing diagnosis from an MGH IPC clinician. When there was no MGH IPC billing diagnosis, we used the billing diagnosis from the child’s pediatrician visit where the MGH IPC referral was made. See Supplementary Fig. S1 for details on the source of billing diagnoses used for MGH IPC analyses.

**S2: Feedback surveys from Young Child Parenting Group and DBT-E**

*Overview*

At the end of each cycle of the Young Child Parenting Group and DBT-E, participants complete anonymous online quality improvement surveys to provide feedback on their experiences in the interventions. These data are used for ongoing quality improvement and to adapt future intervention cycles (e.g., informing updates to inclusion/exclusion criteria, content order, number of sessions, and skills taught). In Supplemental Tables 2-5 below, we present the results from these informal feedback surveys during the study year (July 1, 2023 - June 30, 2024). If a participant did not respond to all questions, we used the data for their completed items (i.e., pairwise deletion). Thus, sample sizes may vary by survey item. Parents were asked to complete the feedback survey during their final virtual group session, and participation was voluntary with no ability to identify respondents. These surveys have shown generally positive responses, with parents in the Young Child Parenting Group self-reporting that the group “moderately” or “extremely” changed their understanding of their child (78.3%), informed their parenting approach (82.6%), and supported them as parents (91.3%). Parents in DBT-E also endorsed significant improvements, self-reporting that DBT-E “moderately” or “extremely” increased their understanding of their child (82.6%), informed their parenting approach (77.3%), and supported them as parents (91.3%). Beyond that, 100% of parents reported that they would recommend DBT-E to other families.

**Table S2** Young Child Parenting Group binary parent survey questions

| **Question item** | **Prevalence** | **Number of responses** |
| --- | --- | --- |
|  | **n (%)** | **n** |
| Children who currently receive Early Intervention services | - | 23 |
| Children who have a current Individualized Education Plan (IEP) or 504 Plan | - | 22 |
| Parents who have already used or plan to use the skills discussed in the group | 23 (100%) | 23 |

Note: - Denotes a frequency (#) of less than 10.

**Table S3** DBT-E binary parent survey questions

| **Question item** | **Prevalence** | **Number of responses** |
| --- | --- | --- |
|  | **n (%)** | **n** |
| Parents who have already used at least one skill discussed in the program | 21 (95.5%) | 22 |
| Parents who would recommend this program to other families | 21 (100%) | 21 |

**Table S4** Young Child Parenting Group Likert-scale parent survey questions

| **Question item** | **Moderately – Extremely** | **Number of responses** |
| --- | --- | --- |
|  | **n (%)** | **n (%)** |
| In the past year, overall, how much did you worry about your child’s emotional and behavioral well-being? | 16 (69.6%) | 23 |
| In the past year, overall, how difficult has it been to find professional behavioral health services for your child? | - | 15 |
| In the past year, overall, how stressful has it been for you to support your child's social, emotional, or behavioral health? | 11 (47.8%) | 23 |
| **Young Child Parenting Group has...** |  |  |
| ...provided useful skills or information | 22 (95.7%) | 23 |
| ...changed my understanding of my child | 18 (78.3%) | 23 |
| ...provided support to me as a parent | 21 (91.3%) | 23 |
| ...informed or changed my parenting approach | 19 (82.6%) | 23 |

Note: - Denotes a frequency (#) of less than 10. As response rates are skewed and cell sizes under n = 10 cannot be reported, the cell sizes for the responses, “Moderately” and “Extremely” were combined to be greater than n = 10.

**Table S5** DBT-E Likert-scale parent survey questions

| **Question item** | **Moderately – Extremely** | **Number of responses** |
| --- | --- | --- |
|  | **n (%)** | **n (%)** |
| In the past year, overall, how difficult  has it been to find professional  behavioral health services for your  child? | 11 (52.4%) | 21 |
| In the past year, overall, how stressful  has it been for you to support your  child's social, emotional, or behavioral  health? | 19 (82.6%) | 23 |
| **DBT-E has...** |  |  |
| ...provided useful skills or information | 19 (86.4%) | 22 |
| ...increased my understanding of my  child | 19 (82.6%) | 23 |
| ...provided support to me as a parent | 21 (91.3%) | 23 |
| ...informed or changed my parenting  approach | 17 (77.3%) | 22 |
| ...improved my ability to manage  my own strong emotions (e.g., when my child is having a tantrum, I considered or used the STOP skill) | 17 (77.3%) | 22 |
| ...improved my child's ability to  manage their strong emotions (e.g.,  calm down faster) | 15 (71.4%) | 21 |
| ...improved my child's behavior  (e.g., less frequent swearing, hitting,  screaming, tantrums; improved  compliance related to daily routines) | 15 (68.2%) | 22 |
| ...improved my relationship with my  child | 17 (81.0%) | 21 |

Note: As response rates are skewed and cell sizes under n = 10 cannot be reported, the cell sizes for the responses “Moderately” and “Extremely” were combined to be greater than n = 10.
